# Silent Culprit Revealed: Renal Infarction Unmasking Atrial Fibrillation

**DOI:** 10.7759/cureus.103934

**Published:** 2026-02-19

**Authors:** Mahima Upadhyay, Susheel Joshi, Mahpara Munir, Omoniyi Akinpeloye, Thair Dawood

**Affiliations:** 1 Internal Medicine, Hurley Medical Center/Michigan State University (MSU), Flint, USA; 2 Internal Medicine, Covenant HealthCare, Saginaw, USA

**Keywords:** atrial fibrillation, dizziness, elderly, flank pain, left flank pain, non valvular atrial fibrillation, renal cortical infarct

## Abstract

Renal infarction is an uncommon yet clinically significant cause of acute flank pain that is frequently misdiagnosed due to its non-specific presentation. Delayed recognition can result in permanent renal damage and missed identification of underlying cardioembolic sources, increasing the risk of recurrent systemic embolization.

We report the case of a 73-year-old man who presented with acute-onset flank pain and intermittent dizziness. Early imaging with contrast-enhanced computed tomography revealed multiple infarcts in the left kidney, confirming the diagnosis of renal infarction. Initial electrocardiography did not demonstrate atrial fibrillation. However, given the absence of an obvious precipitating cause, the patient was admitted for further evaluation and continuous cardiac telemetry monitoring. Subsequent inpatient monitoring revealed paroxysmal atrial fibrillation (PAF), establishing a cardioembolic etiology. Laboratory evaluation initially suggested a hypercoagulable state, which was later attributed to heparin initiation rather than a primary thrombophilic disorder. Anticoagulation therapy with heparin was continued to reduce the risk of further thromboembolic events.

This case underscores the importance of early recognition of renal infarction symptoms and prompt diagnostic imaging. It also highlights the limitations of a single electrocardiographic assessment, as PAF may remain undetected without continuous telemonitoring. Identifying atrial fibrillation as the underlying cause is critical to prevent recurrent embolic complications, including additional renal infarcts or cerebrovascular events. Early hospital presentation, timely imaging, and prolonged cardiac monitoring are essential in unexplained renal infarction to diagnose occult atrial fibrillation and initiate appropriate anticoagulation, thereby preventing further systemic complications.

## Introduction

Renal infarction (RI) is an uncommon but under-recognized condition that may result from thromboembolic phenomena, atrial fibrillation (AF), or intrinsic thrombotic events in hypercoagulable states [1]. Because its symptoms-flank pain, nausea, and sometimes hematuria-overlap with more prevalent conditions such as renal colic and pyelonephritis, diagnosis is often delayed or missed.

Paroxysmal AF is a well-known cause of systemic thromboembolism, especially ischemic stroke. It has also been linked to cognitive decline and dementia, probably because of repeated cerebral hypoperfusion or silent embolic events [2]. Nonetheless, when AF is asymptomatic or occurs sporadically, it may evade detection during routine electrocardiography (ECG) and can be overlooked without extended or continuous cardiac rhythm monitoring [3]. In addition, both inherited and acquired hypercoagulable states independently increase the risk of thrombus formation and systemic embolization, with the risk further amplified when coexisting with cardiac arrhythmias such as AF [4].

## Case presentation

A 73-year-old man with a history of hypertension, prior acute kidney injury, and benign prostatic hyperplasia managed with tamsulosin presented to the emergency department with sudden-onset, severe left flank pain associated with multiple episodes of vomiting and intermittent dizziness. He denied dysuria, hematuria, fever, or other urinary or systemic symptoms.

On arrival, his blood pressure was markedly elevated at 210/114 mmHg, with a heart rate of 88 bpm and oxygen saturation of 98% on room air. Physical examination was notable for pronounced left flank tenderness without signs of peritonitis. Cardiovascular and respiratory examinations were unremarkable.

Initial laboratory evaluation demonstrated leukocytosis of 12.9 × 10⁹/L-attributable to stress response-and an elevated serum creatinine of 2 mg/dL (baseline unknown). Lactate levels were normal. Hemoglobin was 16.2 g/dL, and HbA1c measured 6.6%. Urinalysis revealed glucosuria and ketonuria, with trace hematuria but no pyuria. ECG showed a normal sinus rhythm with no ischemic changes.

**Figure 1:**
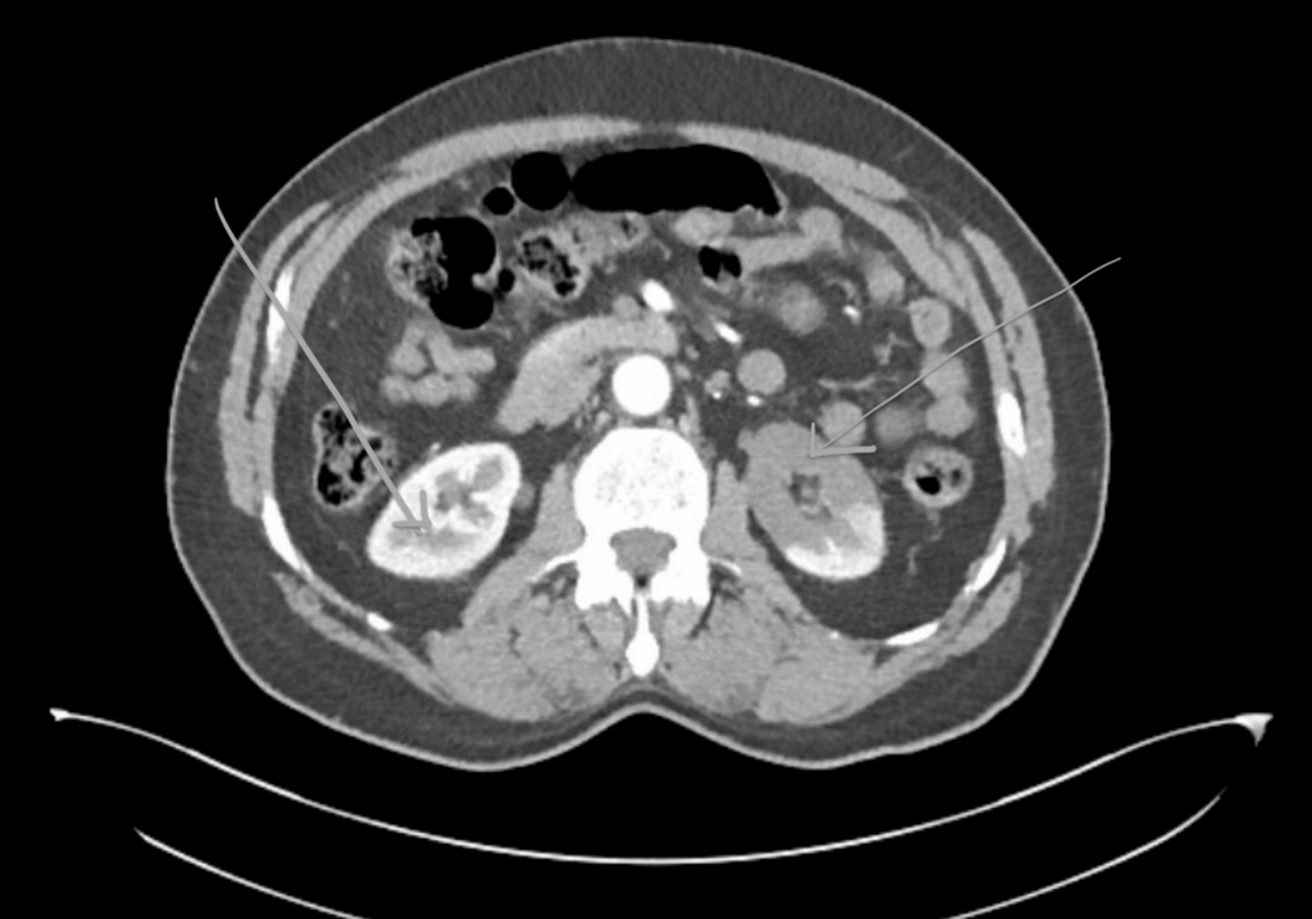
Multifocal wedge-shaped well-demarcated hypoenhancement of the left kidney, no urolithiasis or obstruction

Imaging was pivotal in establishing the diagnosis. Computed tomography (CT) angiography of the abdomen revealed multifocal, wedge-shaped, well-demarcated hypoenhancing lesions in the left kidney, consistent with RIs, with no evidence of urolithiasis or obstructive uropathy (Figure 1). A non-contrast CT confirmed the absence of stones or obstruction. A non-contrast CT of the head revealed no evidence of stroke.

Thrombophilia workup demonstrated elevated homocysteine levels, low antithrombin III, and a positive lupus anticoagulant; however, these results were interpreted with caution given recent heparin initiation (Table 1). During the hospital stay, paroxysmal AF was recorded on telemetry on day 3, establishing a likely cardioembolic etiology.

**Table 1:** Pertinent labs. Coagulable factor elevation in the setting of heparin administration BUN: blood urea nitrogen; PTT: partial thromboplastin time

Labs	Normal	Lab values
White blood cell (WBC) count	4.0-10.8 K/µL	12.9 K/µL
Hemoglobin	13.5-17.5 g/dL	16.2 g/dL
Platelet	130-430 K/µL	315 K/µL
BUN	6-20 mg/dL	24 mg/dL
Creatinine	0.5-1.1 mg/dL	2 mg/dL
Anion gap	3-14 mEq/L	12 mEq/L
Calcium	8.6-10.4 mg/dL	9.6 mg/dL
Urinalysis		2+ protein, no WBC
Prothrombin 20210A analysis	Negative	Negative
Homocysteine day 2	5-13 µmol/L	16.1 µmol/L
Factor 5 day 2	Negative	Negative
Protein C day 2	70%-130%	76%
Protein S day 2	65%-140%	86%
Antithrombin 3 day 2	80%-120%	74%
PTT lupus anticoagulant day 3	<43 s	61 s

**Figure 2:**
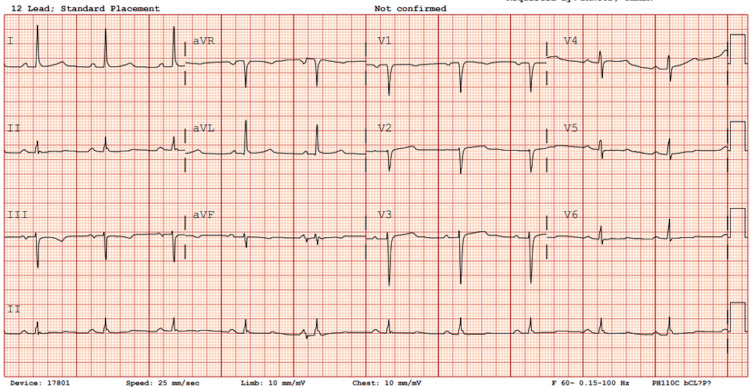
Electrocardiogram status post direct electrical cardioversion restoring normal sinus rhythm, no ischemic changes noted

The patient was anticoagulated with heparin and subsequently underwent successful electrical cardioversion with a 300-joule external biphasic shock, resulting in prompt restoration of sinus rhythm (Figure 2). He was started on metoprolol tartrate 25 mg twice daily, and anticoagulation with heparin was continued with recommendations to maintain therapy for at least six weeks post-cardioversion and indefinitely thereafter, fulfilling the CHA_2_DS_2_VASc score of 3 (age, hypertension, and diabetes) to prevent future risk of thromboembolic events.

Discharge planning included placement of a loop recorder for long-term arrhythmia monitoring and scheduled outpatient follow-up with cardiology and hematology. During the one-month follow-up visit, the patient remained asymptomatic with no recurrent events.

## Discussion

RI accounts for less than 0.1% of emergency department visits but may lead to irreversible kidney damage if not identified promptly [5]. It typically presents with acute flank pain, but the absence of fever, negative urinalysis, and normal imaging for urolithiasis should prompt further evaluation [6]. In this case, the presence of classic wedge-shaped infarcts on CTA allowed early diagnosis and initiation of therapy, averting long-term renal sequelae.

Cardioembolic sources, particularly AF, are leading causes of RI. AF can promote clot formation in the left atrium due to ineffective atrial contraction, leading to blood stasis within the left atrium, particularly the left atrial appendage. This stasis, along with endothelial dysfunction and activation of procoagulant pathways, fulfills Virchow’s triad and creates a prothrombotic state. Thrombi formed in the left atrium can embolize into the systemic circulation, most commonly causing ischemic stroke but also occluding other end-organ arteries. When emboli lodge in the renal arteries, they result in acute interruption of blood flow and subsequent RI, leading to embolism to end organs, including the kidneys [7]. Notably, paroxysmal AF can go undetected on routine ECG, especially when asymptomatic, highlighting the necessity of continuous telemetry monitoring in such patients [8].

In a retrospective study by Hazanov et al., AF was the cause of RI in 64.5% of cases reviewed [6]. Similarly, Kraik et al. emphasized that AF should be suspected in patients with unexplained RI even if the initial ECG is normal [9].

Beyond cardioembolism, hypercoagulable states contribute to RI by promoting in situ thrombosis or enhancing embolic risk. In our case, elevated homocysteine, low antithrombin III, and lupus anticoagulant were present-abnormalities often associated with increased thrombotic risk [10,11]. While lupus anticoagulant can be transiently positive during anticoagulation, repeat testing is warranted.

Studies have shown that both inherited (e.g., antithrombin deficiency and factor V Leiden) and acquired (e.g., antiphospholipid syndrome) thrombophilias are overrepresented in patients with systemic embolism and should be screened in cases lacking an obvious source [12]. This case reinforces the importance of early hospital presentation and comprehensive workup. The prompt use of CTA enabled diagnosis, while telemetry monitoring uncovered the arrhythmic source. Initiation of anticoagulation and subsequent direct current cardioversion restored sinus rhythm and likely prevented further embolic events.

Management of RI often parallels that of other embolic events-urgent anticoagulation is key. Some patients may require interventional procedures such as catheter-directed thrombolysis, thrombectomy, or stenting, but most can be managed conservatively with supportive care and long-term anticoagulation [13,14].

Secondary prevention includes long-term rhythm monitoring, especially for detecting subclinical AF recurrences. Implantable loop recorders can detect intermittent arrhythmias missed by surface ECGs or Holter monitors. Additionally, a stress test and cardiology follow-up before starting antiarrhythmic therapy are standard to rule out structural or ischemic heart disease [15,16]. Despite aggressive treatment, RI can lead to acute kidney injury (AKI), new-onset estimated glomerular filtration rate (eGFR) < 60 mL/min/1.73 m2, end-stage renal disease (ESRD), and death [17].

## Conclusions

RI is a rare but important diagnosis that can be the first sign of hidden AF. Because its presentation often mimics more common causes of flank pain, delayed recognition can lead to preventable renal damage and missed opportunities to identify a systemic embolic source. This case highlights the essential role of early contrast imaging, prolonged cardiac telemetry, and comprehensive coagulation evaluation in patients with unexplained flank pain.

Importantly, this case is distinctive in that the patient initially reported dizziness, which was attributed to tamsulosin therapy. However, continuous inpatient telemetry subsequently revealed paroxysmal AF, establishing a cardioembolic etiology for the RI. This underscores the danger of prematurely attributing non-specific symptoms to medication side effects without thorough evaluation. Subtle or intermittent arrhythmias may otherwise remain undetected, increasing the risk of recurrent embolic events, including stroke or additional organ infarctions. A multidisciplinary and systematic diagnostic approach enables the timely initiation of anticoagulation and targeted management, ultimately reducing long-term renal dysfunction and systemic thromboembolic complications. Early recognition and sustained cardiac monitoring are therefore critical in preventing future morbidity in patients presenting with unexplained RI.
